# Phenotype, genotype and long-term prognosis of 40 Chinese patients with isobutyryl-CoA dehydrogenase deficiency and a review of variant spectra in *ACAD8*

**DOI:** 10.1186/s13023-021-02018-6

**Published:** 2021-09-20

**Authors:** Junqi Feng, Chenxi Yang, Ling Zhu, Yuchen Zhang, Xiaoxu Zhao, Chi Chen, Qi-xing Chen, Qiang Shu, Pingping Jiang, Fan Tong

**Affiliations:** 1 Department of Genetic and Metabolic Disease, The Children’s Hospital, Zhejiang University School of Medicine, National Clinical Research Center for Child Health, 3333 Binsheng Road, Hangzhou, 310052 China; 2 Institute of Genetics and Department of Human Genetics, Zhejiang University School of Medicine, Hangzhou, 310058 China; 3 Zhejiang Provincial Key Lab of Genetic and Developmental Disorders, Hangzhou, 310058 China

**Keywords:** Isobutyryl-CoA dehydrogenase deficiency (IBDD), ACAD8, KMT2A, Genotype, Phenotype, Prognosis

## Abstract

**Background:**

Isobutyryl-CoA dehydrogenase deficiency (IBDD) is a rare autosomal recessive metabolic disorder resulting from variants in *ACAD8*, and is poorly understood, as only dozens of cases have been reported previously. Based on a newborn screening program, we evaluated the incidence, phenotype and genotype of IBDD as well as the prognosis. Moreover, we reviewed the variant spectrum in *ACAD8* associated with IBDD.

**Methods:**

Forty unrelated patients with IBDD were retrospectively screened for newborns between Jan 2012 and Dec 2020. Tandem mass spectrometry (MS/MS) was used to determine the concentrations of C4-acylcarnitine, C4/C2 (acetylcarnitine), and C4/C3 (propionylcarnitine). All suspected cases were genetically tested by metabolic genes panel.

**Results:**

The incidence of IBDD here was 1: 62,599. All patients presented continuously elevated C4-acylcarnitine levels with higher ratios of C4/C2 and C4/C3. Isobutyrylglycine occurred in only 8 patients. During follow-up, four patients had a transient motor delay, and two patients had growth delay. Notably, one case harbored both *ACAD8* compound heterozygous variants and a *KMT2A *de novo variant (c.2739del, p.E914Rfs*35), with IBDD and Wiedemann–Steiner syndrome together, had exact severe global developmental delay. All patients were regularly monitored once they were diagnosed, and each patient gradually had a normal diet after 6 months of age. After 3–108 months of follow-up, most individuals were healthy except the case harboring the *KMT2A* variant. A total of 16 novel variants in *ACAD8*, c.4_5delCT, c.109C > T, c.110–2A > T, c.236G > A, c.259G > A, c.381–14G > A, c.413delA, c.473A > G, c.500delG, c.758 T > G, c.842–1G > A, c.911A > T, c.989G > A, c.1150G > C, c.1157A > G and c.1165C > T, were identified. Along with a literature review on 51 *ACAD8* variants in 81 IBDD patients, we found that the most common variant was c.286G > A (27.2%), which has been observed solely in the Chinese population to date, followed by c.1000C > T (8.6%), c.1176G > T (3.7%) and c.455 T > C (3.1%).

**Conclusion:**

The concentration of C4-acylcarnitine in NBS plus subsequent genetic testing is necessary for IBDD diagnosis. Both the genotypes and ACAD8 variants in IBDD are highly heterogeneous, and no significant correlations between genotype and phenotype are present here in patients with IBDD. Our IBDD cohort with detaied clinical characteristics, genotypes and long-term prognosis will be helpful for the diagnosis and management of patients with IBDD in the future.

**Supplementary Information:**

The online version contains supplementary material available at 10.1186/s13023-021-02018-6.

## Background

Isobutyryl-CoA dehydrogenase (IBD) deficiency (IBDD, OMIM#611283) is a rare autosomal recessive metabolic disorder involving defects in valine metabolism [1], which is caused by biallelic variants in *ACAD8* (acyl-CoA dehydrogenase family member 8; OMIM*604773) on chromosome 11q25 [2]. IBD is a mitochondrial enzyme that functions as a soluble homotetramer to catalyse the conversion of isobutyryl-CoA to methacrylyl-CoA in the degradation of the branched chain amino acid (BCAA)—‘valine’, which finally converges on the tricarboxylic acid cycle. To date, only dozens of IBDD cases have been reported worldwide since it was first described in 1998, with an estimated prevalence of 1/45,466 in southern Italy and 1/292,451 in California [3, 4]. Affected individuals are mostly diagnosed through newborn screening (NBS) and characterized by elevated C4-acylcarnitine levels, with or without secondary carnitine deficiency [1, 5–8]. However, elevated urine isobutyrylglycine (IBG) levels have been documented as additional supporting evidence, but this change is not always present in patients with IBDD [9]. Patients with IBDD are reported to be either asymptomatic [5, 10], or symptomatic with variable clinical features, including failure to thrive, seizures, anaemia, muscular hypotonia, and developmental delay [5–9, 11, 12]. Some symptomatic patients had normal growth and development with carnitine supplementation and avoidance of fasting to limit amino acid metabolism during childhood [5]. In contrast, one asymptomatic child with IBDD was reported to develop clinical symptoms, including muscle pain, muscle weakness and tiredness, in adulthood [13], indicating a complicated pathogenesis and suggesting that patients with IBDD should be monitored carefully [14]. As elevated C4-acylcarnitine concentrations are neither specific nor sufficient for the clinical diagnosis of IBDD, genetic testing is increasingly applied for the identification of IBDD. Recently, more than 30 variants in *ACAD8* have been reported in different ethnic populations. However, the clinical, genetic and prognostic data of IBDD are limited by the number of cases. Here, we retrospectively summarized the phenotypes, genotypes and long-term prognosis of up to 40 patients with IBDD identified between 2012 and 2020, and reviewed all *ACAD8* variants in described IBDD cases, including 16 novel variants in this study.

## Materials and methods

### Subjects

Forty patients with a diagnosis of IBDD based on the NBS program within 7 days after birth were retrospectively reviewed between 2012 and December 2020 in a single site newborn screening program, among which 30 patients were identified from 1,877,970 infants between January 2017 and December 2020. This study was conducted at the Children’s Hospital, Zhejiang University School of Medicine, and approved by the Research Ethics Committees. Patient information was tabulated in this article without individual patient identifiers.

### Metabolic marks analysis

Tandem mass spectrometry (MS/MS) was used to determine the concentrations of C4-acylcarnitine, C4/C2 (acetylcarnitine), and C4/C3 (propionylcarnitine) with dried blood spot filter paper samples in the NBS program. Urine samples were collected from the patients for a urine organic acid analysis by gas chromatograph-mass spectrometer (GC–MS). The following cut-off parameters in our clinical lab were used for validation: normal values of C4-acylcarnitine ranged from 0.03 to 0.48 (μmol/L) and those of urinary IBG ranged from 0 to 0.4 (mmol/mol). Moreover, the normal C4/C2 ratio decreased in the 0–0.05 interval, and the C4/C3 ratio decreased in the 0.04–0.46 interval. The Wechsler Intelligence Scale for Children-R (WISC-R) or the Ages-Stages Questionnaire (ASQ) were used to assess patients' developmental status. Length/height-for-age and weight-for-age standards are according to China growth standards (0–3 years old) and WHO Child Growth Standards (> 3 years old).

### Genetic analysis

Triometabolic gene panel tests by NGS (next-generation sequencing) were conducted in all suspected cases who were picked up by NBS, including the *ACAD8, ACADS, ETHE1, ETFA, ETFB, and ETFDH* genes. The DNA library was prepared using an Agilent SureSelect Inherited Disease Capture Kit (Agilent, USA) and sequenced using an Illumina MiSeq platform (Illumina, USA). The quality of the DNA library was tested with a Qubit and 2100 Bioanalyzer (Agilent High Sensitivity DNA Kit, Agilent). The sequencing libraries were quantified using an Illumina DNA Standards and Primer Premix Kit (Kapa, USA). The paired-end reads were quality trimmed using the Trimmomatic program (http://www.usadellab.org/cms/index.php?page¼trimmomatic) and aligned with the human genome reference sequence (UCSC Genome build hg19). Single-nucleotide polymorphisms (SNPs) and insertions or deletions were identified using the Samtools program (http://www.htslib.org/). The ClinVar and Human Gene Mutation Database (HGMD) was used to search for known pathogenic mutations. Automatic tools (SIFT, Polyphen, MutationTaster, CADD, etc.) were used to predict the functional significance of novel variants. Variants were classified according to the recommendations of the American College of Medical Genetics and Genomics (ACMG) as follows: pathogenic (P), likely pathogenic (LP), variants of unknown significance (VUS), likely benign (LB), and benign (B) [15].

## Results

### Clinical manifestations and interventions

A cohort of 40 unrelated patients with IBDD is summarized in Table 1. The IBDD incidence was 1: 62,599 based on data from the NBS program between 01/2017 and 12/2020. In this cohort, patients #9 and #22 were born prematurely, but their mothers’ pregnancies were uneventful to that point, and they did not require any significant interventions. The remaining 38 patients were born after uneventful pregnancies at full term. All 40 patients were evaluated for significantly increased concentrations of C4-acylcarnitine ranging from 0.98 to 3.36 μmol/L and elevated C4/C2 and C4/C3 ratios by NBS. Consistent with previous documents, isobutyrylglycine occurred in only 8 patients (patients #2, #11, #13, #16, #21, #27, #35 and #38) from the cohort. Ten patients (patients #1, #12, #19, #25, #27, #29, #31, #36, #39 and #40) had anaemia as described elsewhere [10]. Some biochemical indicators associated with liver function, such as aminotransferase and gamma-glutamyl transferase, were aberrant in 18 patients from 0.5–7 months after birth. However, the relationship between abnormal liver function and IBDD is not clear as abnormal liver function is common in most children patients with inherited metabolic disease, especially in infants. Regarding development, patients #9, #10, #12 and #14 experienced a transient motor delay, and all recovered during follow-up. However, patient #33 had a severe developmental delay, with an FIQ of 53 according to WISC-R. Subsequently, by whole exome sequencing and analysis, a *de nov*o variant c.2739del (p.E914Rfs*35) in *KMT2A* was detected in patient #33, which was absent in gnomAD (Genome Aggregation Database). As *KMT2A* is known as the causative gene in Wiedemann-Steiner syndrome (WDSTS, OMIM#605130), this truncating variant was implied to be responsible for the manifestations of severe growth and developmental delay in patient #33. The height/length-for-age and weight-for-age of patients #5, #15, #17, #19 and #22 were slightly delayed during follow-up. Patient #25, who with milk allergy, and patient #33 had severe thriving failure.

**Table 1:** Genotypes and clinical features in 40 IBDD cases

Case	Genotype		Pathogenicity	CADD Score		NBS			Clinical
	Allele 1	Allele 2	classification	Allele 1	Allele 2	C4 (0.03–0.48 µmol/L)	C4/C2 (0–0.05)	C4/C3 (0.04–0.46)	symptom
1	c.4_5delCT^#^	c.842-1G > A^#^	P/P	20.7	31	1.86 ↑	0.16 ↑	1.14 ↑	④
	(p.L2Vfs*40)								
2	c.286G > A	c.286G > A	LP/LP	30	30	1.79 ↑	0.11 ↑	1.28 ↑	③
	(p.G96S)	(p.G96S)							
3	c.286G > A	c.1176G > T	LP/LP	30	25.5	1.5 ↑	0.11 ↑	1.02 ↑	–
	(p.G96S)	(p.R392S)							
4	c.1000C > T	c.235C > G	LP/LP	27.2	24.7	1.2 ↑	0.1 ↑	1.5 ↑	–
	(p.R334C)	(p.R79G)							
5	c.286G > A	c.286G > A	LP/LP	30	30	1.24 ↑	0.05	0.98 ↑	④
	(p.G96S)	(p.G96S)							
6	c.286G > A	c.911A > T^#^	LP/VUS	30	31	1.57 ↑	0.13 ↑	0.86 ↑	–
	(p.G96S)	(p.Q304L)							
7	c.286G > A	–	LP/?	30	–	1.06 ↑	0.07 ↑	0.7 ↑	④
	(p.G96S)								
8	c.286G > A	c.455 T > C	LP/LP	30	27.4	2.55 ↑	0.18 ↑	2.48 ↑	–
	(p.G96S)	(p.M152T)							
9	c.286G > A	c.712delT	LP/P	30	35	1.66 ↑	0.21 ↑	3.07 ↑	②
	(p.G96S)	p.W238 fs*9							
10	c.286G > A	c.286G > A	LP/LP	30	30	1.66 ↑	0.1 ↑	1.71 ↑	②④
	(p.G96S)	(p.G96S)							
11	c.1000C > T	c.286G > A	LP/ LP	27.2	30	1.86 ↑	0.15 ↑	1.84 ↑	③④
	(p.R334C)	(p.G96S)							
12	c.1000C > T	c.286G > A	LP/ LP	27.2	30	2.02 ↑	0.09 ↑	1.64 ↑	②④
	(p.R334C)	(p.G96S)							
13	c.286G > A	c.235C > G	LP/LP	30	24.7	2.88 ↑	0.13 ↑	1.18 ↑	③④
	(p.G96S)	(p.R79G)							
14	c.286G > A	c.444G > T	LP/LB	30	4.199	1.16 ↑	0.04	0.56 ↑	②
	(p.G96S)	(p.P148P)							
15	c.286G > A	c.413delA^#^	LP/P	30	23.4	1.67 ↑	0.1 ↑	1.01 ↑	④
	(p.G96S)	(p.N138Mfs*36)							
16	c.286G > A	c.286G > A	LP/LP	30	30	2.96 ↑	0.11 ↑	1.55 ↑	③
	(p.G96S)	(p.G96S)							
17	c.413delA^#^	c.500delG^#^	P/P	23.4	25.3	1.59 ↑	0.11 ↑	1.92 ↑	–
	(p.N138Mfs*36)	(p.S167Mfs*7)							
18	c.286G > A	c.286G > A	LP/LP	30	30	1.8 ↑	0.09 ↑	0.81 ↑	–
	(p.G96S)	(p.G96S)							
19	c.110-2A > T^#^	c.109C > T#	P/LP	32	23.8	1.86 ↑	0.1 ↑	2.21 ↑	④
		(p.P37S)							
20	c.1176G > T	c.444G > T	LP/LB	25.5	4.199	0.98 ↑	0.05	0.58 ↑	–
	(p.R392S)	(p.P148P)							
21	c.286G > A	c.286G > A	LP/LP	30	30	1.43 ↑	0.08 ↑	0.88 ↑	③④
	(p.G96S)	(p.G96S)							
22	c.259G > A^#^	c.1000C > T	VUS/LP	27.1	27.2	1.9 ↑	0.23 ↑	2.84 ↑	④
	(p.G87R)	(p.R334C)							
23	c.500delG#	c.758 T > G#	P/VUS	25.3	28.5	3.36 ↑	0.12 ↑	0.85 ↑	–
	(p.S167Mfs*7)	(p.V253G)							
24	c.1176G > T	c.1176G > T	LP/LP	25.5	25.5	2.33 ↑	0.07 ↑	1.08 ↑	–
	(p.R392S)	(p.R392S)							
25	c.1157A > G^#^	c.1000C > T	VUS/LP	23.8	27.2	1.09 ↑	0.07 ↑	1.38 ↑	①④
	(p.Q386R)	(p.R344C)							
26	c.4_5delCT#	C.617G > A	P/LP	20.7	30	2.29 ↑	0.11 ↑	1.89 ↑	–
	(p.L2Vfs*39)	(p.R206Q)							
27	c.989G > A^#^	c.381-14G > A^#^	LP/VUS	27.9	6.957	1.59 ↑	0.08 ↑	1.31 ↑	③④
	(p.R330Q)								
28	c.1000C > T	c.617G > A	LP/ LP	27.2	30	2.57 ↑	0.14 ↑	1.82 ↑	④
	(p.R344C)	(p.R206Q)							
29	c.286G > A	c.286G > A	LP/ LP	30	30	1.64 ↑	0.06 ↑	1.39 ↑	④
	(p.G96S)	(p.G96S)							
30	c.286G > A	c.286G > A	LP/ LP	30	30	2.09 ↑	0.14 ↑	1.53 ↑	–
	(p.G96S)	(p.G96S)							
31	c.236G > A^#^	c.286G > A	LP/LP	27.2	30	0.99 ↑	0.13 ↑	1.15 ↑	④
	(P.R79Q)	(p.G96S)							
32	c.413delA^#^	c.286G > A	P/ LP	23.4	30	1.83 ↑	0.29 ↑	3.33 ↑	④
	(p.N138Mfs*36)	(p.G96S)							
33*	c.286G > A	c.1000C > T	LP/ LP	30	27.2	1.97 ↑	0.09 ↑	1.99 ↑	①②④
	(p.G96S)	(p.R344C)							
34	c.286G > A	c.1150G > C^#^	LP/VUS	30	21.3	1.04 ↑	0.11 ↑	1.24 ↑	④
	(p.G96S)	(p.V384L)							
35	c.473A > G^#^	c.413delA^#^	VUS/P	31	23.4	1.82 ↑	0.19 ↑	2.28 ↑	③④
	(p.Y158C)	(p.N138Mfs*36)							
36	c.1000C > T	c.1092 + 1G > A	LP/P	27.2	33	1.9 ↑	0.1 ↑	1.2 ↑	④
	(p.R344C)								
37	c.286G > A	c.500delG^#^	LP/P	30	25.3	1.61 ↑	0.08 ↑	1.46 ↑	–
	(p.G96S)	(p.S167Mfs*7)							
38	c.286G > A	c.500delG^#^	LP/P	30	25.3	1.43 ↑	0.08 ↑	0.48 ↑	③④
	(p.G96S)	(p.S167Mfs*7)							
39	c.286G > A	c.286G > A	LP/LP	30	30	2.42 ↑	0.07 ↑	0.9 ↑	④
	(p.G96S)	(p.G96S)							
40	c.286G > A	c.1165C > T^#^	LP/LP	30	26.5	2.71 ↑	0.13 ↑	1.12 ↑	④
	(p.G96S)	(p.R389W)							

Clinical symptom includes ①growth delay, ②motor delay, ③elevated urine IBG, ④abnormal other blood biochemical parameters; “–”: normal

*IBD* isobutyryl-CoA dehydrogenase; *NBS* Newborn screening; *C4* isobutyl carnitine; *C2* acetyl carnitine; *C3* propionyl carnitine; *LP* likely pathogenic; *P* pathogenic; *VUS* variant uncertain significance; *LB* likely benign

^#^Novel variants in this study

33*, patient #33 have a de novo variant c.2739del (p.E914Rfs*35) in *KMT2A*,

Consistent with published cases, most patients with IBDD turned out healthy and had no clinical sequelae [6, 10, 16]. Once diagnosed, all patients with IBDD were treated with sufficient caloric supplementation and dietary high-protein restriction (protein intake: 2–2.5 g/kg/d) to avoid fasting and avoid protein catabolism. Under the guidance of medical specialists, infants younger than 6 months were nourished with full breastfeeding or formula supplementation when breast milk was insufficient. Elder infants were nourished additionally with introduced complementary foods to meet daily protein requirements. The feeding interval for infants less than 6 months was no more than 4 h, that for infants 6–12 months was no more than 6–8 h, that for infants 1–3 years was no more than 8–10 h, and that for infants 3 years and above was no more than 10–12 h. Patient #33 accepted L-carnitine (50 mg/kg/d) and vitamin B2 (100 mg/d) supplementation for one year and then stopped since there was no improvement in clinical symptoms or biochemical indictors. Patient #25 was diagnosed with cow's milk protein allergy (CMPA) by a gastroenterologist, and then his complementary food was changed to extensively hydrolysed formula, which has insufficient calories to supply and may account for his growth delay during infancy. Fortunately, his growth delay was improved later with the diet transitioning to toddler nutrition. Moreover, each parent had been educated and reminded to introduce complementary foods as stated and then gradually transitioned to normal toddler nutrition after 1.5–2 years of age. After 6 months of follow-up, each patient gradually had a normal diet. During follow-up, each patient’s height/length, weight and health were regularly monitored. Assisted by familial rehabilitation, no other metabolic imbalance occurred, while C4-acylcarnitine was still sustained at high concentrations (Additional file 1: Table S2).

### Genetic variants and genotypes

**Fig. 1:**
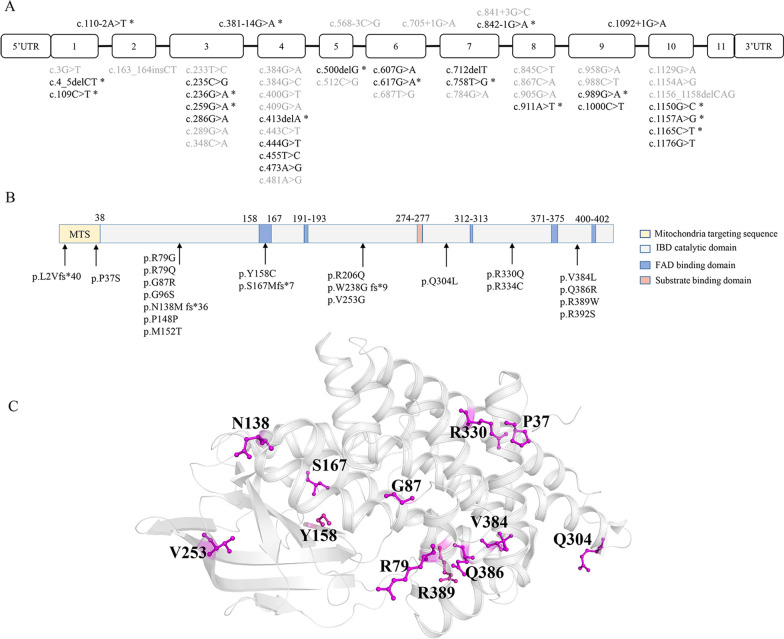
Twenty-five *ACAD8* variants were identified in 40 patients with IBDD. **A** Twenty-five variants distributed in both the exons and introns of *ACAD8*. Black, variants detected in this study; grey, variants reported previously; *, novel variants identified in this study. **B** Distribution of 21 variants in the IBD protein domain, without the intron variants. **C** 12 novel variants in protein 3D-structure. Three novel splicing variants in introns and the MTS variant p. L2Vfs*40 were not illustrated. Referenced sequences for the *ACAD8* gene, protein and structure are NM_014384.2, HUMAN NP_055199.1, and PDB entry, 1RX0, respectively

Biallelic variants were genotyped in 39 individuals except patient #7, in which only one variant was detected (Table 1). Twenty-nine (74%) patients carried compound heterozygous variants, and 10 (26%) patients carried homozygous variants. The predominant homoallelic genotype was [p. G96S];[p. G96S], detected in 9 individuals, and the other one was [p. R392S];[ p. R392S] in patient #24. As shown in Fig. 1A, 25 variants were identified in our patients, including 4 splicing variants (16%; c.110–2A > T, c.381–14G > A, c.842–1G > A, c.1092 + 1G > A), a synonymous variant (c.444G > T p. P148P), 16 missense variants (64%), and 4 truncating variants (16%) resulting from 4 deletions, c.4_5delCT (p. L2Vfs*40), c.413delA (p. N138Mfs*36), c.500delG (p. S167Mfs*7) and c.712delT (p. W238fs*9). Given its protein structure [17], most amino acid alterations caused by variants in IBD are localized in its catalytic domain (Fig. 1B), while two variants, p. L2Vfs*40 and p. P37S are located in the mitochondria targeting sequence (MTS), and the p.S167Mfs*7 variant occurs in a binding domain for substrate FAD (flavin adenine dinucleotide). Notably, 16 novel variants were identified, including 10 missense variants (Fig. 1C): c.109C > T (p. P37S), c.236G > A (p. R79Q), c.259G > A (p. G87R), c.473A > G (p. Y158C), c.758 T > G (p. V253G), c.911A > T (p. Q304L), c.989G > A (p. R330Q), c.1150G > C (p. V384L), c.1157A > G (p. Q386R), and c.1165C > T (p. R389W); 3 truncating variants: c.4_5delCT (p. L2Vfs*40), c.413delA (p. N138Mfs*36) and c.500delG (p. S167Mfs*7); and 3 splicing variants described above.

According to ACMG recommendations (Table 1), three novel truncating variants (c.4_5delCT, c.413delA, c.500delG) and novel splicing variants (c.110–2A > T, c.381–14G > A, c.842–1G > A) were postulated to be pathogenic (P), as along with the reported pathogenic variant c.455 T > C. The novel variant c.236G > A alters an arginine to glutamine (p. R79Q), like the previously reported c.235C > G (p. R79G) variant [10]. Similarly, the novel variant c.989G > A changes the same amino acid, R330, which was previously reported in c.988C > T (R330W) [7]. As shown in Fig. 1B, both c.236G > A and c.989G > A are localized in their catalytic domains, while c.109C > T is in the mitochondria-targeting sequence, which is crucial for protein imported into mitochondria. Novel missense variants c.911A > T and c.1157A > G were absent in the gnomAD database. Based on the evaluation using bioinformatics programs (Additional file 1: Table S1), all 10 novel missense variants were predicted as functional variants that would exert damaging effects on its protein.

The missense variant c.286G > A (p. G96S) was reported to be most common in the Chinese population, with an approximately frequency of 50% [10], which occurred here in 26 individuals with an allelic frequency of 44%, followed by c.1000C > T (p. R334C), c.1176G > T (p. R392S), c.413delA (p. N138Mfs*36) and c.500delG (p. S167Mfs*7) with allelic frequencies of 10%, 5%, 5% and 5%, respectively. Most patients had their unique genotype. There were 27 genotypes distributed in 39 biallelic individuals. Except for the [c.286G > A];[ c.286G > A] genotype in 9 patients, there were 3 genotypes shared by more than two patients, followed as [c.286G > A]; [c.1000C > T] by 3 patients (P#11, #12 and #33), [c.286G > A]; [c.413delA] by 2 patients (P#15 and #32), and [c.286G > A]; [c.500delG] by 2 patients (P#37 and #38).

### Review of *ACAD8* variants in IBDD

**Fig. 2:**
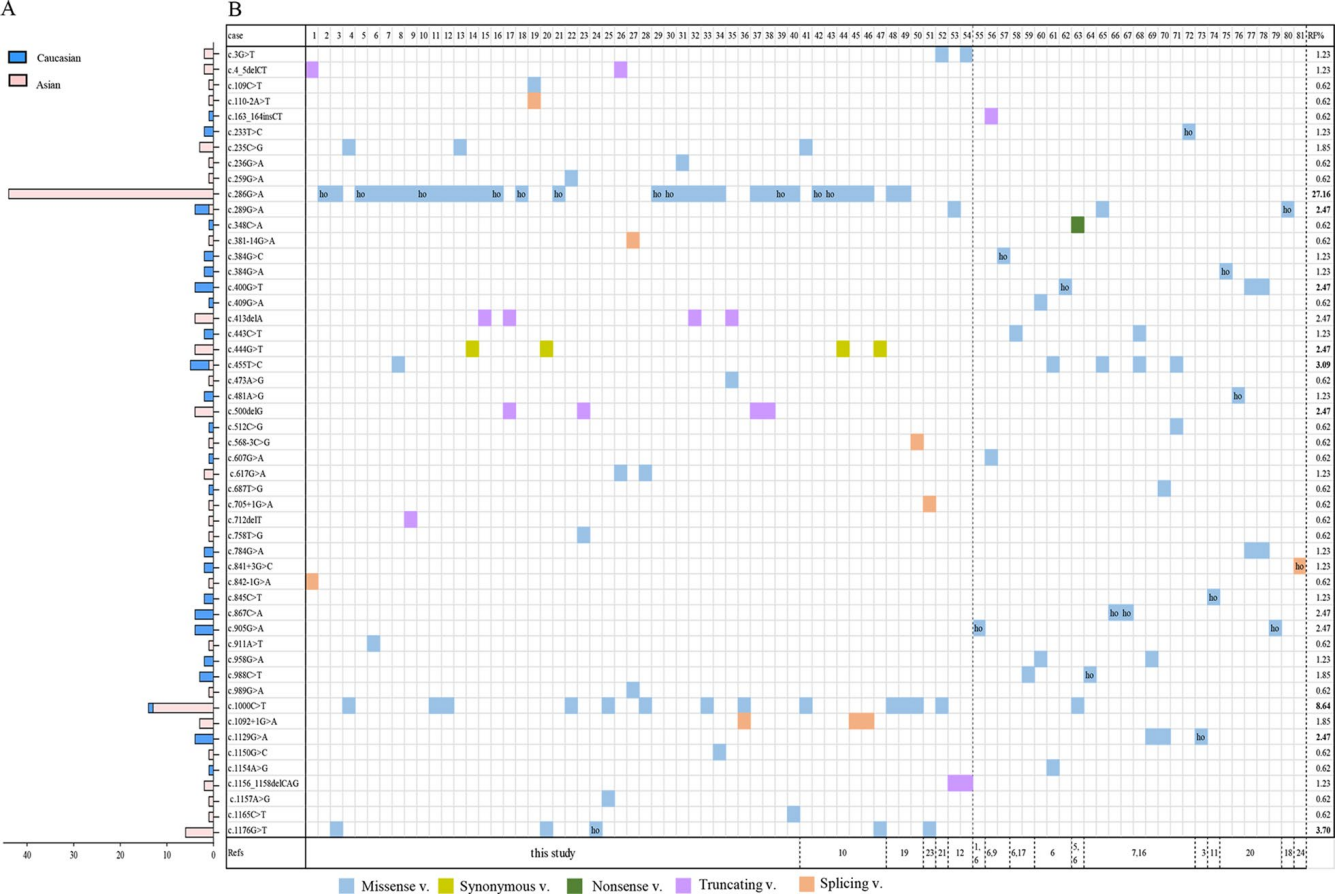
Overview of the *ACAD8* variants contributed to 81 IBDD patients. **A** *ACAD8* variants in cases and different populations. **B** The genotypes and relative allelic frequency in IBDD patients. *ho* homozygous; *RF* relative allelic frequency in the 81 patients’ group; *Refs* references. *Note* Cases 1–55 were reported in the Asian population; Cases 56–81 were reported in the Caucasian population

Including our patient, 87 individuals with IBDD have been described [1–7, 9–23], of which 81 underwent genetic testing for *ACAD8* and 78 individuals were confirmed to have biallelic variants. Fifty-one types of variants were detected, including 36 (70%) missense variants, one nonsense variant (2%, c.348C > A), one synonymous variant (2%, c.444G > T), 7 (14%) splicing sites between introns and exon barriers and 6 (12%) truncating variants. As shown in Fig. 2A, only 3 variants, c.289G > A, c.455 T > C and c.1000 C > T, occurred in both Asian and Caucasian population. To date, the variant c.286 G > A was most prevalent in IBDD, specifically present in 44 Chinese patients [10, 19], followed by variants c. 1000C > T in 14 patients, c.1176G > A in 6 patients and c. 455 T > C in 5 patients. There were 57 different genotypic combinations in 78 patients. Consistent with our finding, 67% of cases with biallelic variants were predominantly genotyped with compound heterozygous alleles (Fig. 2B). However, only 26 IBDD individuals carried homozygous alleles (Fig. 2B). Similarly, most patients (62%, 48/78) had their unique genotype. However, 9 different genotypic combinations were shared in 30 individuals, including [c.286G > A];[c.286G > A] in 9 patients and [c.286G > A]; [c.1000C > T] in 5 patients (Fig. 2B).

## Discussion

IBDD is a rare metabolic disorder that is less well understood, as only dozens of cases have been described previously in the literature [10–12]. Due to the limited number of cases, the IBDD prevalence varies substantially in different regions and/or populations. Here, based on the NBS program and clinical diagnosis, 40 patients with IBDD were identified with a prevalence of 1/62,599 in Zhejiang Province, which is close to the incidence (1/70,000) estimated by Oglesbee et al. [7], but higher than the incidence reported in California (1/292,451) by Gallant et al. [4]. Consistent with published cases, most patients with IBDD turned out healthy and had no clinical sequelae. Clinical symptoms, including increased urinary IBG levels, mild delay in growth and development, were generally transient during follow-up and had been restored by dietary management. However, there was no direct correlation between the improvement of clinical outcomes (including growth-developmental delay and impaired liver function) and diet, and several developmental delay cases followed an age-appropriate intake of calories and protein after 6 months of age, similar to other children. Nevertheless, elevated C4-acylcarnitine concentrations always exist. Neonatal hypoglycemia in patients with IBDD that reported elsewhere was absent here [20], while 10 patients had anaemia instead. However, *Acad8* mutant mice showed significantly elevated transaminase levels and presented progressive hepatic steatosis [24]. Strikingly, some biochemical indicators associated with liver function were aberrant in 17 patients during follow-up. Regarding anaemia or transient liver lesions, we are still unaware whether those clinical symptoms are in connection with IBDD. A few patients had developmental delay, speech delay or hypotonia [10, 12]. Unfortunately, patient #33 presented a severe delay in both growth and development with an inflexible increase in serum C4-acylcarnitine levels, which was further genetically confirmed to be a novel variant, c.2739del (p. E914Rfs*35), in *KMT2A* that associated with WDSTS. Another special IBDD patient, with a severe lack of speech development and lack of social interactions, was reported to be associated with autism that was genetically confirmed in the *DNA2* gene [18]. Therefore, appropriate interventions are required once IBDD is diagnosed, and patients with IBDD should be monitored carefully. All patients received proper dietary interventions as stated and had a positive prognosis during clinical monitoring.

Both the genotypes and *ACAD8* variants in IBDD are highly heterogeneous. A total of 51 variants were reported in 81 IBDD patients and were widely distributed along the gene. More than 60% of patients presented with a unique genotype, and the compound heterozygotes were predominant. The variants c.286G > A (p. G96S), c.1000C > T (p. R334C), c.1176G > A (p. R392S) and c.455 T > C (p. M152T) appeared to be more prevalent in 81 IBDD patients, with relative frequencies of 27.2%, 8.6%, 3.7% and 3.1% (Fig. 2B). To date, the hotspot variant, c.286G > A has been observed solely in Chinese patients [10, 19], indicating that it is specific in this population and needs more cases to be confirmed. In this study, 16 *ACAD8* variants were first reported in IBDD patients. It should be certain that more novel variants will be detected in future cases with the widespread utilization of genetic testing in IBDD. The genotype–phenotype correlation was unclear here. Nine patients with the same genotype, [c.286G > A];[c.286G > A], presented mild symptoms or were asymptomatic with different biochemical indicators (Additional file 1: Table 2). On the other hand, 8 patients with elevated urinary IBG carried different genotypes, including LP/LP, LP/P or P/VUS combination (Table 1). Intriguingly, Patients #14 and #20, harboring a synonymous variant c.444G > T (p. P148P) with a low CADD score [25], had a normal level of C4/C2 (0.05) at newborn screening and occasionally elevated to 0.08 during follow-up. Patient #17, with 2 deletions, potentially no IBD activity, had moderate elevations of C4 without a phenotype.

In summary, up to 40 patients with IBDD were diagnosed with a prevalence of 1/62,597 in Zhejiang Province. IBDD patients picked up by NBS have a mild phenotype here. Most patients were healthy during follow-up, except one who was associated with WDSTS. Sixteen novel *ACAD8* variants were identified. Based on a review of the variant spectrum in IBDD, we found that c.286G > A and c.1000C > T were prevalent in patients, of which c.286G > A has been observed solely in the Chinese population. Similar to other metabolic disorders, compound heterozygotes were predominant in genotypes. No clear genotype–phenotype correlation existed in IBDD patients. Here, our IBDD cohort with detailed clinical characteristics, genotypes and long-term prognosis will be helpful for the diagnosis and management of patients with IBDD.

## Conclusions

Isobutyryl-CoA dehydrogenase deficiency is a rare metabolic disorder with an incidence of 1/62,599 in Zhejiang Province. The concentration of C4-acylcarnitine in NBS plus subsequent genetic testing is helpful for IBDD diagnosis. Both the genotypes and ACAD8 variants in IBDD are highly heterogeneous, No clear correlation between genotype and phenotype is presented here in patients with IBDD.

## Supplementary Information

**Additional file 1: Table 1**. redicted functional effects of 10 novel missense variants; **Table 2**. Clinical features and genotypes of 40 IBDD patients.

## Data Availability

The dataset supporting the conclusions of this article is included within the article and its supplementary information files.
