# Correction to: Phenotype, genotype and long-term prognosis of 40 Chinese patients with isobutyryl-CoA dehydrogenase deficiency and a review of variant spectra in ACAD8

**DOI:** 10.1186/s13023-021-02132-5

**Published:** 2021-12-07

**Authors:** Junqi Feng, Chenxi Yang, Ling Zhu, Yuchen Zhang, Xiaoxu Zhao, Chi Chen, Qi-xing Chen, Qiang Shu, Pingping Jiang, Fan Tong

**Affiliations:** 1 Department of Genetic and Metabolic Disease, The Children’s Hospital, Zhejiang University School of Medicine, National Clinical Research Center for Child Health, 3333 Binsheng Road, Hangzhou, 310052 China; 2 Institute of Genetics and Department of Human Genetics, Zhejiang University School of Medicine, Hangzhou, 310058 China; 3 Zhejiang Provincial Key Lab of Genetic and Developmental Disorders, Hangzhou, 310058 China

## Correction to: Orphanet J Rare Dis (2021) 16:392 10.1186/s13023-021-02018-6

Following the publication of the original article [1] the authors asked to revise the following sentence of the “Discussion” section:

"Another special IBDD patient, with a severe lack of speech development and lack of social interactions, was reported to be associated with autism that was genetically confirmed in the *DNA2* gene [18]."

The correct sentence should read:

"Another special IBDD patient, with a severe lack of speech development and lack of social interactions, was reported to be associated with autism [18]."

The original article has already been corrected as above.
