# Supplementary material for: Phenotype, genotype and long-term prognosis of 40 Chinese patients with isobutyryl-CoA dehydrogenase deficiency and a review of variant spectra in ACAD8
Source: Orphanet J Rare Dis. 2021 Sep 20;16:392. doi: 10.1186/s13023-021-02018-6 (PMC8454130; doi:10.1186/s13023-021-02018-6)
Supplement: Supplementary file 1 — Additional file 1: Table 1. redicted functional effects of 10 novel missense variants; Table 2. Clinical features and genotypes of 40 IBDD patients. [file 13023_2021_2018_MOESM1_ESM.pdf]

Supplementary Table 1. Predicted functional effects of 10 novel missense variants

**Variants**	**CADD**	**SIFT**	**Polyphen2_HDIV**	**LRT**	**MutationTaster**	**FATHMM**
c.109C>T, p. P37S	23.8	D	P	D	A	D
c.236G>A, p. R79Q	27.2	T	P	D	D	D
c.259G>C, p. G87R	27.1	D	D	D	D	D
c.473A>G, p. Y158C	31	D	D	D	A	D
c.758T>G, p. V253G	28.5	D	B	D	D	D
c.911A>T, p. Q304L	31	D	D	D	D	D
c.989G>A ,p. R330Q	27.9	D	D	D	D	D
c.1150G>C ,p. V384L	21.3	T	P	D	D	D
c.1157A>G, p. Q386R	23.8	T	B	D	D	D
c.1165C>T, p. R389W	26.5	D	D	D	A	D

A, Automaticallydisease-causing; B, Benign; D, Deleterious or Damaging; P, possibly damaging; T, Tolerated.

Supplementary Table 2. Clinical features and genotypes of 40 IBDD patients.

Patient	1	2	3	4	5	6	7	8	9
Gender	Male	male	male	male	male	female	male	female	female
Nucleotide change	c.4_5delCT	c.286G>A	c.286G>A	c.1000C>T	c.286G>A	c.286G>A	c.286G>A	c.286G>A	c.286G>A
	c.842-1G>A	c.286G>A	c.1176G>T	c.235C>G	c.286G>A	c.911A>T	-	c.455T>C	c.712delT
Amino acid change	p.L2Vfs*40	p.G96S	p.G96S	p.R334C	p.G96S	p.G96S	p.G96S	p.G96S	p.G96S
	-	p.G96S	p.R392S	p.R79G	p.G96S	p.Q304L	-	p.M152T	p.W238 fs
ACMG classification	P/P	LP/LP	LP/LP	LP/LP	LP/LP	LP/VUS	LP/-	LP/LP	LP/P
Gestations(weeks)	38	38	38	38	40	39	38	40	35
Birth weight(kg)	3.3	3.5	3.02	3.8	3.85	3.2	2.9	2.65	2.4
NBS age (days)	3	3	4	3	4	5	4	14	17
NBS	↑C4	↑ C4	↑ C4	↑ C4	↑ C4	↑ C4	↑ C4	↑ C4	↑ C4
C4 (umol/l)	1.86	1.79	1.50	1.20	1.24	1.57	1.06	2.55	1.66
C4/C2	0.16	0.11	0.11	0.10	0.05	0.13	0.07	0.18	0.21
C4/C3	1.14	1.28	1.02	1.50	0.98	0.86	0.70	2.48	3.07
Age at last f/u	1y	7m	3y1m	1y9m	5y	4m	1y10m	3y2m	2y5m
C4 range(umol/l)	1.79-2.5	1.09-1.79	0.62-1.81	1.18-2.06	0.73-1.54	1.57-2.38	0.57-2.29	1.56-2.66	1.66-2.61
PMH	-	-	Transient hematuria	-	liver function lesion	-	-	-	Gross motor delay
Elevated urine IBG	No	Yes	No	No	No	No	No	No	No
Growth delay	No	No	No	No	mild	No	No	No	No
Developmental delay	No	No	No	No	No	No	No	No	Yes
Blood biochemistry	Hb ↓	Normal	Normal	Normal	bile acid ↑	Normal	Prealbumin ↓	Normal	Normal
					Prealbumin ↓				
					ALT ↑				
					AST ↑				
					LAC ↑				
					Blood zinc ↓				
					Bilirubin ↑				

Table S2 continued

Patient	10	11	12	13	14	15	16	17	18
Gender	Male	male	male	male	male	male	female	female	male
Nucleotide change	c.286G>A<br>c.286G>A	c.1000C>T<br>c.286G>A	c.1000C>T<br>c.286G>A	c.286G>A<br>c.235C>G	c.286G>A<br>c.444G>T	c.286G>A<br>c.413delA	c.286G>A<br>c.286G>A	c.413delA<br>c.500delG	c.286G>A<br>c.286G>A
Amino acid change	p.G96S<br>p.G96S	p.R334C<br>p.G96S	p.R334C<br>p.G96S	p.G96S<br>p.R79G	p.G96S<br>p.P148P	p.G96S<br>p.N138Mfs*36	p.G96S<br>p.G96S	p.N138Mfs*36<br>p.S167Mfs*7	p.G96S<br>p.G96S
ACMG classification	LP/LP	LP/ LP	LP/ LP	LP/ LP	LP/ LB	LP/ P	LP/ LP	P/ P	LP/ LP
Gestations(weeks)	40	38	39	39	40	39	37	39	39
Birth weight(kg)	3.35	3.1	3.47	3.86	3.56	3.1	2.81	2.93	3.25
NBS age (days)	4	4	3	4	3	3	4	3	4
NBS	↑C4	↑C4	↑C4	↑C4	↑C4	↑C4	↑C4	↑C4	↑C4
C4 (umol/l)	1.66	1.86	2.02	2.88	1.16	1.67	2.96	1.59	1.8
C4/C2	0.1	0.15	0.09	0.13	0.04	0.1	0.11	0.11	0.09
C4/C3	1.71	1.84	1.64	1.18	0.56	1.01	1.55	1.92	0.81
Age at last f/u	1y9m	1y1m	2y	4m	1y11m	1y	1y9m	1y7m	5m
C4 range(umol/l)	0.94-1.8	0.94-1.86	0.8-2.02	1.11-2.88	0.47-1.22	1.17-1.98	1.59-2.96	0.95-2.96	1.04-1.8
PMH	Gross motor delay	-	Gross motor/ social delay	-	Gross motor delay	-	-	-	-
Elevated urine IBG	No	Yes	No	Yes	No	No	Yes	No	No
Growth delay	No	No	No	No	No	mild	No	mild	No
Developmental delay	Yes	No	Yes	No	Yes	No	No	No	No
Blood biochemistry	Blood zinc ↓<br>TC ↑<br>CK ↑	bile acid ↑<br>Prealbumin ↓<br>GGT ↑<br>Bilirubin ↑	Prealbumin ↓<br>ALT ↑<br>AST ↑<br>Blood zinc ↓<br>Bilirubin ↑<br>Hb ↓<br>ALP ↑	Bilirubin ↑<br>ammonia ↑	Normal	bile acid ↑<br>Prealbumin ↓<br>ALT ↑<br>AST ↑<br>LAC ↓	Normal	Normal	Normal

Table S2 continued

Patient	19	20	21	22	23	24	25	26	27
Gender	Male	male	male	female	female	male	male	male	male
Nucleotide change	c.110-2A>T	c.1176G>T	c.286G>A	NA	c.500delG	c.1176G>T	c.1157A>G	c.4_5delCT	c.989G>A
	c.109C>T	c.444G>T	c.286G>A	c.1000C>T	c.758T>G	c.1176G>T	c.1000C>T	C.617G>A	c.381-14G>A
Amino acid change	-	p.R392S	p.G96S	p.G87R	p.S167Mfs*7	p.R392S	p.Q386R	p.L2Vfs*39	p.R330Q
	p.P37S	p.P148P	p.G96S	p.R334C	p.V253G	p.R392S	p.R334C	p.R206Q	-
ACMG classification	P/ LP	LP/ LB	LP/ LP	VUS/LP	P/VUS	LP/LP	VUS/LP	P/LP	LP/LP
Gestations(weeks)	40	38	38	36	39	39	37	39	40
Birth weight(kg)	2.89	3.65	3.95	2.15	3.45	2.95	2.58	4.45	3.9
NBS age (days)	3	3	4	11	3	3	4	3	3
NBS	↑ C4	↑ C4	↑ C4	↑ C4	↑ C4	↑ C4	↑ C4	↑ C4	↑ C4
C4 (umol/l)	1.86	0.98	1.43	1.9	3.36	2.33	1.09	2.29	1.59
C4/C2	0.1	0.05	0.08	0.23	0.12	0.07	0.07	0.11	0.08
C4/C3	2.21	0.58	0.88	2.84	0.85	1.08	1.38	1.89	1.31
Age at last f/u	1y7m	6m	1y1m	11m	3m	4m	5m	4m	5m
C4 range(umol/l)	1.08-2.73	0.47-0.98	1.22-1.83	1.02-2.46	1.39-3.36	1.09-2.33	0.79-1.18	1.14-2.29	1.08-1.89
PMH	-	-	-	-	-	-	-	-	-
Elevated urine IBG	No	No	Yes	No	No	No	No	No	Yes
Growth delay	Mild	No	No	mild	No	No	mild	No	No
Developmental delay	No	No	No	No	No	No	No	No	No
Blood biochemistry	LAC ↑	Normal	bile acid ↑	bile acid ↑	Normal	Normal	ALT ↑	Normal	LAC ↑
	Hb ↓			TG ↑			GGT ↑		Hb ↓
	ALP ↑						Blood zinc ↓		

Table S2 continued

Patient	28	29	30	31	32	33	34
Gender	Female	male	male	female	female	male	male
Nucleotide change	c.1000C>T<br>c.617G>A	c.286G>A<br>c.286G>A	c.286G>A<br>c.286G>A	c.236G>A<br>c.286G>A	c.413delA<br>c.286G>A	c.286G>A<br>c.1000C>T	c.286G>A<br>c.1150G>C
Amino acid change	p.R344C<br>p.R206Q	p.G96S<br>p.G96S	p.G96S<br>p.G96S	P.R79Q<br>p.G96S	p.N138Mfs*36<br>p.G96S	p.G96S<br>p.R344C	p.G96S<br>p.V384L
ACMG classification	LP/ LP	LP/ LP	LP/ LP	LP/ LP	P/ LP	LP/ LP	LP/ VUS
Gestations(weeks)	38	38	40	40	38	37	39
Birth weight(kg)	3.11	3.4	3.29	3.22	3.3	2.9	2.87
NBS age (days)	3	4	3	3	4	4	3
NBS	↑C4	↑C4	↑C4	↑C4	↑C4	↑C4	↑C4
C4 (umol/l)	2.57	1.64	2.09	0.99	1.83	1.97	1.04
C4/C2	0.14	0.06	0.14	0.13	0.29	0.09	0.11
C4/C3	1.82	1.39	1.53	1.15	3.33	1.99	1.24
Age at last f/u	10m	10m	1m	2m	4m	7y5m	5y12m
C4 range(umol/l)	1.24-2.57	0.99-2.59	1.76-2.07	0.99-1.53	1.81-2.18	1.08-1.97	0.69-2.26
PMH	-	-	-	-	Abnormal fatty acid metabolism	Wiedemann-Steiner syndrome<br>Urine ethylmalonic acid ↑	Urine 3-hydroxyisobutyric acid ↑
Elevated urine IBG	No	No	No	No	No	No	No
Growth delay	No	No	No	No	No	severe	No
Developmental delay	No	No	No	No	No	severe	No
Blood biochemistry	LAC ↑<br>ammonia ↑	Hb ↓<br>ammonia ↑	Normal	Prealbumin ↓<br>bile acid ↑<br>Hb ↓<br>ammonia ↑	AST ↑<br>bile acid ↑<br>TG ↑<br>CK ↑	LAC ↑<br>Prealbumin ↓<br>TG ↑	AST ↑<br>CK ↑<br>TG ↑

Table S2 continued

Patient	35	36	37	38	39	40
Gender	Male	female	male	female	male	male
Nucleotide change	c.473A>G	c.1000C>T	c.286G>A	c.286G>A	c.286G>A	c.286G>A
	c.413delA	c.1092+1G>A	c.500delG	c.500delG	c.286G>A	c.1165C>T
Amino acid change	p.Y158C	p.R334C	p.G96S	p.G96S	p.G96S	p.G96S
	p.N138Mfs*36	-	p.S167Mfs*7	p.S167Mfs*7	p.G96S	p.R389W
ACMG classification	LP/ P	LP/ P	LP/ P	LP/P	LP/LP	LP/LP
Gestations(weeks)	38	38	39	38	38	37
Birth weight(kg)	3.26	1.78	4.0	3.84	3.9	3.37
NBS age (days)	3	30	3	3	3	4
NBS	↑ C4	↑ C4	↑ C4	↑ C4	↑ C4	↑ C4
C4 (umol/l)	1.82	1.90	1.61	1.43	2.42	2.71
C4/C2	0.19	0.10	0.08	0.08	0.07	0.13
C4/C3	2.28	1.20	1.46	0.48	0.9	1.12
Age at last f/u	3m	2m	1m	5m	3m	2m
C4 range(umol/l)	1.78-2.16	1.9-2.78	1.61-2.34	1.43-1.92	1.65-2.42	2.1-2.71
PMH	-	-	-	-	-	-
Elevated urine IBG	Yes	No	No	Yes	No	No
Growth delay	No	No	No	No	No	No
Developmental delay	No	No	No	No	No	No
Blood biochemistry	LAC ↑	LAC ↑	Normal	CK ↑	bile acid ↑	LAC ↑
	ammonia ↑	ammonia ↑		GGT ↑	TG ↑	ammonia ↑
		Hb ↓		Prealbumin ↓	Hb ↓	Hb ↓
					Prealbumin ↓	

NBS: newborn screening; C4: C4-acylcarnitine; C2: acetyl carnitine; C3: propionyl carnitine; f/u: follow up; PMH: past medical history; IBG: isobutyrylglycin;

Blood biochemistries include Bile acid, Blood zinc, Prealbumin, ammonia, ALT, AST, GGT, LAC, CK, ALP, Bilirubin, TC/TG and Hemoglobin.

ALT: alanine aminotransferase; AST: aspartate aminotransferase; GGT: gamma-glutamyltransferase; LAC: lactic acid; CK: creatine kinase; ALP: alkaline phosphatase; Hb: hemoglobin; TC: total cholesterol; TG: triglyceride. ↑ increased; ↓ decreased.
